# Effects of Ultrasound-Guided Superficial Cervical Plexus Block With Clavipectoral Fascial Plane Block Versus Superficial Cervical Plexus Block With Interscalene Brachial Plexus Block in Clavicle Surgery: A Prospective Study

**DOI:** 10.7759/cureus.99388

**Published:** 2025-12-16

**Authors:** Ganapathy Srinivasan, Vinod Krishnagopal, Raj Murugan

**Affiliations:** 1 Anaesthesiology, Sree Balaji Medical College and Hospital, Chennai, IND

**Keywords:** brachial plexus, clavicle, nerve block, postoperative pain, regional anesthesia, ultrasonography

## Abstract

Background

Perioperative pain control in clavicle fixation is challenging due to complex innervation and limitations of conventional anesthesia. Regional techniques provide safer, opioid-sparing alternatives. This study compared the efficacy and safety of superficial cervical plexus block with clavipectoral fascial plane block versus interscalene brachial plexus block in clavicle fixation surgery.

Methodology

In this prospective randomized study, 60 patients scheduled for open reduction and internal fixation of clavicle fractures were allocated into two groups of 30 each. Group 1 received superficial cervical plexus block combined with clavipectoral fascial plane block (SCPB+CPB), while Group 2 received superficial cervical plexus block combined with interscalene brachial plexus block (SCPB+ISBPB). Primary outcomes included duration of analgesia and time to first rescue analgesic requirement. Secondary outcomes were postoperative pain scores, total analgesic consumption, hemodynamic stability, and complications. Data were analyzed using standard statistical tests (chi-square) by SPSS software version 27 (IBM Corp., Armonk, NY, USA). A p-value <0.05 was considered significant.

Results

The SCPB+CPB group demonstrated significantly longer analgesia (14.6 ± 2.1 hours vs. 10.3 ± 1.9 hours, p < 0.001) and delayed rescue analgesic demand (13.8 ± 2.0 hours vs. 9.6 ± 1.8 hours, p < 0.001). Pain scores were consistently lower in Group 1 at all postoperative intervals. Total analgesic consumption within 24 hours was significantly reduced in Group 1. Complications such as hemidiaphragmatic paralysis occurred only in Group 2.

Conclusions

SCPB+CPB provides comparatively better analgesia, reduces rescue analgesic use, and avoids respiratory complications compared to SCPB+ISBPB, establishing it as a safe and effective anesthetic technique for clavicle fixation surgery.

## Introduction

Clavicle fractures are common skeletal injuries, comprising about 2.6% of all fractures and 44% of shoulder girdle injuries [1], most often occurring in the structurally weaker middle third due to trauma or falls [2]. They cause significant pain, functional impairment, and cosmetic deformity, with some requiring surgical fixation [3]. Perioperative pain management remains challenging due to the variable innervation of the clavicle, involving both the superficial cervical plexus (C3-C4) and branches of the brachial plexus [4]. General anesthesia, though effective, is linked to nausea, hemodynamic instability, delayed recovery, and opioid-related adverse effects, prompting a shift toward regional anesthesia and multimodal, opioid-sparing strategies [5].

Regional anesthesia offers surgical anesthesia, reduced opioid use, hemodynamic stability, and faster recovery, with ultrasound guidance improving precision [6]. The interscalene brachial plexus block (ISBPB), the traditional choice, provides reliable analgesia but may not cover the first postoperative night and carries risks such as phrenic nerve palsy and respiratory complications [7]. The superficial cervical plexus block (SCPB) alone is inadequate for deeper analgesia [8]. The clavipectoral fascial plane block (CPB) targets deeper innervation while avoiding phrenic nerve involvement [9], and combining SCPB with CPB may achieve complete analgesia.

Evidence comparing SCPB+CPB with SCPB+ISBPB remains limited, though early studies support CPB’s effectiveness [10]. This study strives to address this gap by comparing their analgesic duration, opioid requirements, and hemodynamic effects. We hypothesize that SCPB+CPB offers superior pain control and fewer complications, guiding safer, more effective anesthetic strategies for clavicle surgery.

The primary objectives of the study are to compare the duration of postoperative analgesia between the two regional anesthetic techniques over a 24-hour postoperative period and to compare the time to first rescue analgesic requirement between the study groups within 24 hours after surgery. The secondary objectives of the study are to compare postoperative pain intensity using Visual Analog Scale (VAS) scores at 30 minutes, 4, 8, 12, 18, and 24 hours postoperatively, to compare the total rescue analgesic consumption within the first 24 postoperative hours, to evaluate and compare intraoperative and postoperative hemodynamic and respiratory parameters between the two groups during the observation period, and to assess and compare the incidence of block-related complications between the two techniques.

## Materials and methods

Study design and setting

This prospective, comparative, observational study was conducted in the Department of Anaesthesiology at Sree Balaji Medical College and Hospital, Chennai, a tertiary care center with advanced orthopedic and trauma services. The study compared two ultrasound-guided regional anesthetic techniques, namely, superficial cervical plexus block with clavipectoral fascial plane block (SCPB+CPB) and SCPB with interscalene brachial plexus block (SCPB+ISBPB), for clavicle fixation surgeries.

Study period

The study was conducted over 18 months (January 2023-June 2024). Interim reviews ensured protocol adherence and consistency in data collection.

Study population

Elective and emergency patients with clavicular fractures scheduled for open reduction and internal fixation during the study period were enrolled and randomized into two groups to receive either superficial cervical plexus block combined with clavipectoral fascial plane block (SCPB+CPB) or superficial cervical plexus block combined with interscalene brachial plexus block (SCPB+ISBPB).

Inclusion criteria

Group 1 (SCPB+CPB)

Adults aged 18-65 years with American Society of Anesthesiologists (ASA) physical status I or II, undergoing unilateral clavicle fracture fixation under ultrasound-guided SCPB combined with CPB, who provided written informed consent.

Group 2 (SCPB+ISBPB)

Adults aged 18-65 years with ASA physical status I or II, undergoing unilateral clavicle fracture fixation under ultrasound-guided SCPB combined with ISBPB, who provided written informed consent.

Exclusion criteria

Patients with local infection, coagulopathy, allergy to local anesthetics, significant cardiopulmonary disease, pre-existing neurological deficits, phrenic nerve palsy, pregnancy, polytrauma, or refusal to consent were excluded.

Sampling technique and sample size

A prospective, comparative observational sampling strategy was used. All eligible patients undergoing unilateral clavicle fracture fixation during the study period were screened consecutively, and those fulfilling the inclusion criteria were enrolled until the required sample size was achieved.

The sample size was fixed at 60 patients (30 in each group) based on feasibility and the expected patient volume during the study period. A minimum of 25 patients per group was considered adequate to detect clinically meaningful differences in analgesic duration based on effect sizes reported in previously published literature. The final sample size was increased to 30 patients per group to compensate for potential exclusions due to protocol deviations or incomplete data recording.

Procedure

Group Allocation

Group allocation was determined by the regional anesthetic technique administered as part of routine clinical practice, based on anesthesiologist preference and patient suitability. Patients receiving ultrasound-guided SCPB+CPB were included in Group 1, while those receiving SCPB+ISBPB were included in Group 2. No randomization was performed.

Block Performance

All regional blocks were performed under ultrasound guidance by consultant anesthesiologists with more than five years of experience in regional anesthesia, following standardized institutional protocols.

Block Technique and Perioperative Sedation

All regional blocks were performed preoperatively in a dedicated block area under standard monitoring (electrocardiography, non-invasive blood pressure, and pulse oximetry). Blocks were administered under strict aseptic precautions using a high-frequency linear ultrasound probe by consultant anesthesiologists with more than five years of experience in ultrasound-guided regional anesthesia.

SCPB was performed using an in-plane ultrasound-guided technique at the level of the C4 vertebra, depositing local anesthetic in the superficial cervical plexus plane beneath the sternocleidomastoid muscle.

For patients in Group 1 (SCPB+CPB), a clavipectoral fascial plane block was performed under ultrasound guidance by depositing local anesthetic between the clavipectoral fascia and the periosteum of the clavicle at the medial and lateral aspects of the fracture site.

For patients in Group 2 (SCPB+ISBPB), an interscalene brachial plexus block was performed under ultrasound guidance at the level of the C5-C6 nerve roots using the standard interscalene approach.

Local Anaesthetic Drug and Dose

For both groups, 0.25% bupivacaine was used as the local anesthetic agent: SCPB: 10 mL, CPB: 15 mL (Group 1), and ISBPB: 15 mL (Group 2). The total dose of bupivacaine did not exceed the maximum recommended safe dose (2.5 mg/kg). Negative aspiration was performed before each injection, and incremental injection with continuous ultrasound visualization was ensured in all cases.

Intraoperative Sedation and Analgesia Protocol

Intraoperative sedation was provided only when required for patient comfort. Sedation was achieved using intravenous midazolam (0.02-0.05 mg/kg) and/or intravenous fentanyl (0.5-1 µg/kg) as titrated doses, maintaining spontaneous respiration and verbal responsiveness. No routine intraoperative systemic analgesics were administered unless patients reported discomfort. Adequacy of surgical anesthesia was confirmed before incision based on the absence of pain on surgical stimulation and hemodynamic stability. No patient required conversion to general anesthesia.

Safety Measures

Standard precautions to prevent local anesthetic systemic toxicity were followed, including continuous monitoring, ultrasound guidance, avoidance of intravascular injection, and availability of lipid emulsion therapy in the operating room. Patients were continuously monitored intraoperatively and postoperatively for neurological, respiratory, and hemodynamic complications.

Data collection

Data Collection Tool and Procedure

Data were collected using a structured, predesigned case record form. Baseline demographic details, intraoperative hemodynamic parameters (heart rate, systolic and diastolic blood pressure), and respiratory parameters were recorded at predefined intervals. Postoperative pain was assessed using the Visual Analog Scale (VAS score = 0-10). VAS scores were recorded at 30 minutes, 4 hours, 8 hours, 12 hours, 18 hours, and 24 hours postoperatively. Hemodynamic and respiratory parameters were documented intraoperatively at baseline, after block performance, and at regular intraoperative intervals, as well as postoperatively during the 24-hour observation period.

Outcome Measures

The primary outcomes were duration of analgesia (defined as time from completion of regional block to first request for rescue analgesia) and time to first rescue analgesic administration. Secondary outcomes included postoperative VAS scores at predefined intervals, total rescue analgesic consumption within 24 hours, intraoperative and postoperative hemodynamic and respiratory stability, and block-related complications.

Statistical analysis

Data were analyzed using SPSS software version 27 (IBM Corp., Armonk, NY, USA). Normally distributed continuous variables were expressed as mean ± standard deviation and compared between groups using the independent samples t-test. Categorical variables were expressed as frequencies and percentages and analysed using the chi-square test.

The primary outcomes, i.e., duration of analgesia and time to first rescue analgesic, were compared between groups using the independent samples t-test, and results were presented along with mean differences and 95% confidence intervals (CIs). Repeated-measures analysis of variance was used to evaluate within-group changes over time and between-group differences for repeated measurements, including postoperative VAS pain scores and serial hemodynamic parameters such as heart rate and blood pressure.

All statistical tests were two-tailed, and a p-value less than 0.05 was considered statistically significant. Corresponding 95% CIs were calculated wherever applicable to indicate the precision of estimates.

Ethical considerations

Institutional ethics approval was obtained from Sree Balaji Medical College and Hospital (approval number: 002/SBMCH/IHEC/2023/2064). Written informed consent was secured, and confidentiality was maintained in accordance with the Declaration of Helsinki.

## Results

**Figure 1:**
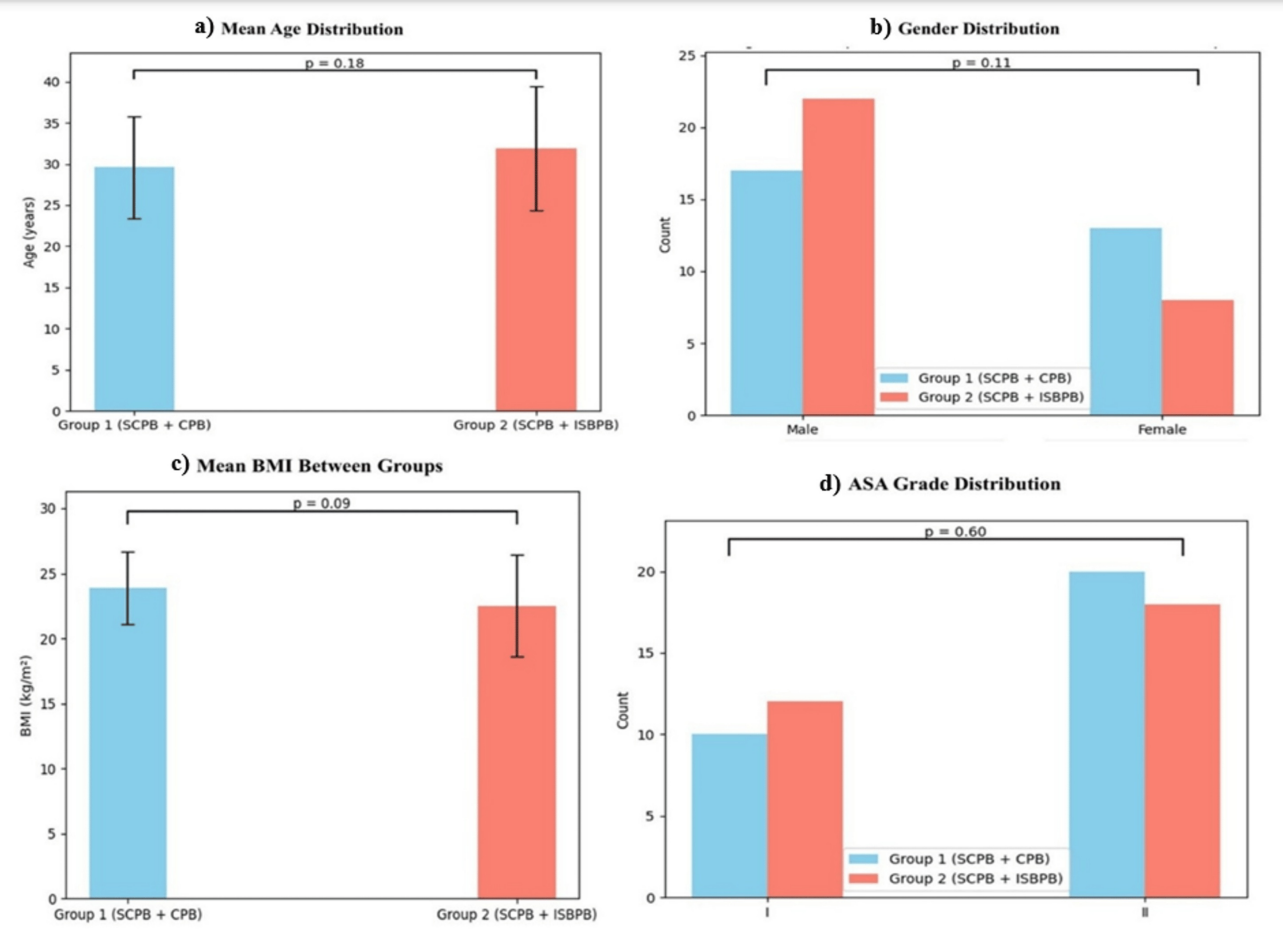
Distribution of age, gender, BMI, and ASA grade between experimental groups (N = 30). An independent samples t-test was used for continuous variables (age, BMI), and the chi-square test was used for categorical variables (gender, ASA) P-value <0.05 is statistically significant. ASA = American Society of Anesthesiologists; BMI = body mass index; SCPB+CPB = superficial cervical plexus block combined with clavipectoral fascial plane block; SCPB+ISBPB = superficial cervical plexus block combined with interscalene brachial plexus block

A total of 60 patients were enrolled and randomly allocated into two equal groups: Group 1 (SCPB+CPB) and Group 2 (SCPB+ISBPB). Demographic variables were well balanced, minimizing potential confounding effects. The mean ± SD age was 36.8 ± 9.4 years in Group 1 and 37.2 ± 10.1 years in Group 2, with no statistically significant difference (p > 0.05). Gender distribution was comparable, with 22 males and 8 females in Group 1, and 21 males and 9 females in Group 2. The body mass index (mean ± SD) was 24.1 ± 2.7 kg/m² in Group 1 and 24.4 ± 2.9 kg/m² in Group 2 (p > 0.05). Most patients were ASA I, with 23 in Group 1 and 22 in Group 2, and the remainder were ASA II (Figure 1).

**Figure 2:**
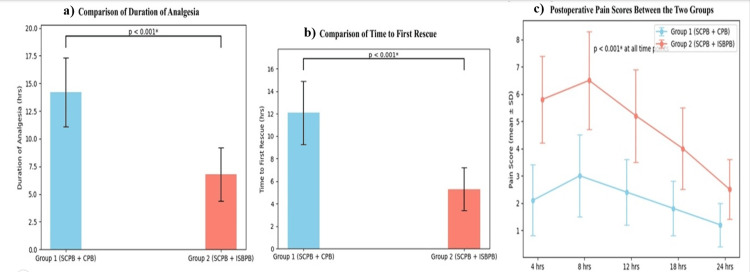
Comparison of duration of analgesia, time to first rescue, and VAS scores (N = 30). Independent samples t-test was used for each continuous variable between groups, and repeated-measures analysis of variance was used for the analysis of VAS at multiple time points. *: p-value <0.05 is statistically significant. VAS = Visual Analog Scale; SCPB+CPB = superficial cervical plexus block combined with clavipectoral fascial plane block; SCPB+ISBPB = superficial cervical plexus block combined with interscalene brachial plexus block

A primary outcome was the duration of analgesia, defined as the time from block completion to first analgesic requirement. Group 1 (SCPB+CPB) had a significantly longer duration (14.6 ± 2.1 hours) than Group 2 (SCPB+ISBPB) (10.3 ± 1.9 hours, p < 0.001). The mean difference was 4.3 hours with a 95% CI of 3.3 to 5.3 hours (p < 0.001). Similarly, the time to first rescue analgesic was delayed in Group 1 (13.8 ± 2.0 hours) versus Group 2 (9.6 ± 1.8 hours, p < 0.001). The mean difference was 4.2 hours with a 95% CI of 3.2 to 5.2 hours (p < 0.001). Postoperative pain scores (VAS) were minimal in both groups at baseline and 30 minutes. However, differences became significant at later intervals. At 4 hours, VAS scores were 2.1 ± 0.6 in Group 1 and 2.8 ± 0.7 in Group 2; at 8 hours, 3.4 ± 0.8 vs. 4.6 ± 1.0; and at 12 hours, 4.1 ± 0.9 vs. 5.7 ± 1.1. Group 1 maintained significantly lower scores even at 18 and 24 hours (p < 0.001) (Figure 2).

**Figure 3:**
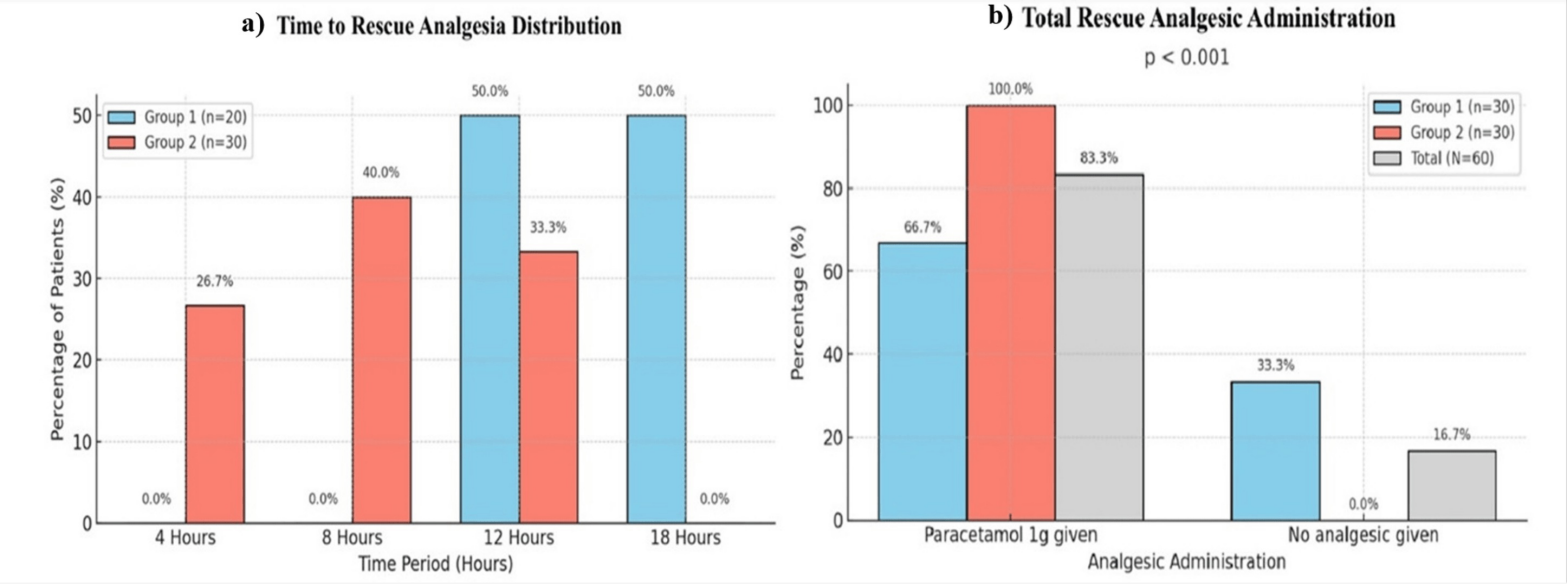
Comparison of time to rescue analgesia distribution between the two groups and total rescue analgesic administration (N = 30). The chi-square was test used for categorical distribution, and the t-test was used for the mean number of doses. *: p-value <0.05 is statistically significant.

Rescue analgesic use was significantly lower in Group 1. The mean number of doses in 24 hours was 1.2 ± 0.5 in Group 1 compared to 2.1 ± 0.6 in Group 2 (p < 0.001). Most Group 1 patients (70%) required only one dose, whereas 60% of Group 2 patients required multiple doses (Figure 3).

**Figure 4:**
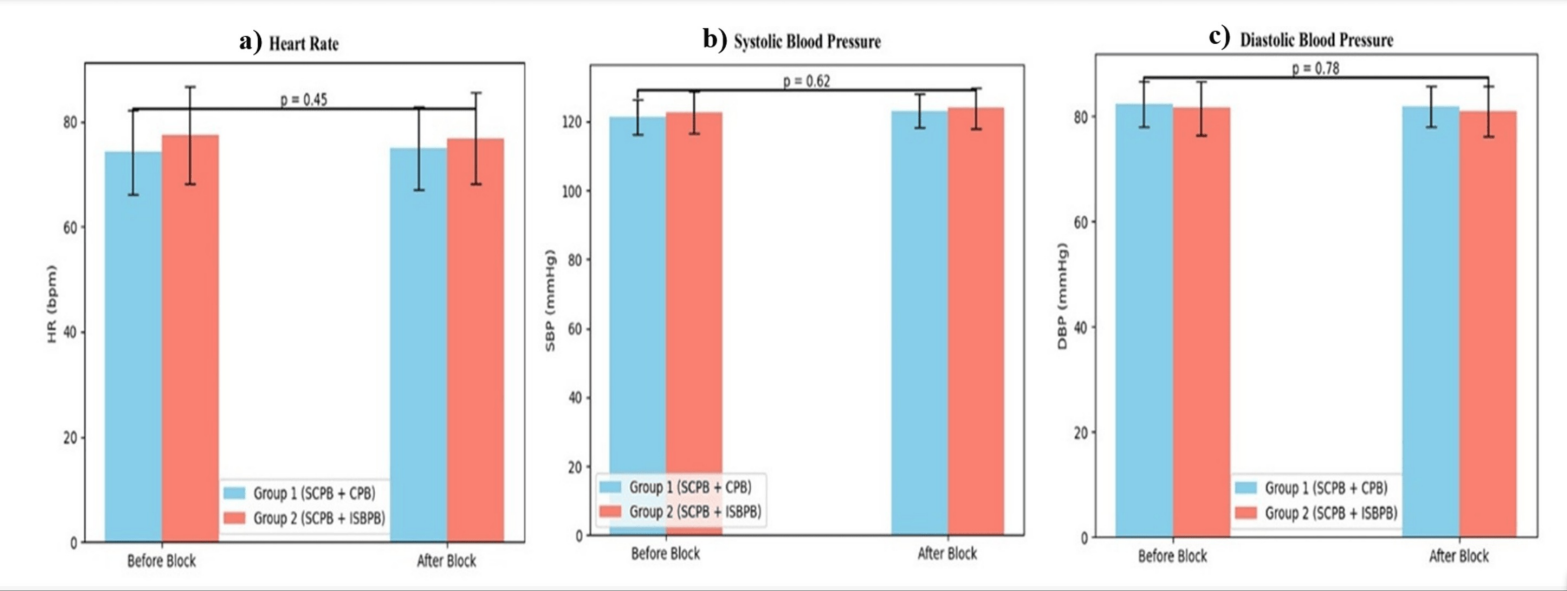
Comparison of heart rate and blood pressure (N = 30). (a) Comparison of heart rate (beats/minute in n). (b) Comparison of systolic blood pressure (mmHg in n). (c) Comparison of diastolic blood pressure (mmHg in n). The independent samples t-test was used for group comparison at each time point, and the repeated-measures analysis of variance was used for the analysis of time trends. P-value <0.05 is statistically significant.

Hemodynamic parameters remained stable in both groups. Mean heart rate ranged from 70-88 beats/minute in Group 1 and 72-94 beats/minute in Group 2. Systolic blood pressure ranged from 112-126 mmHg and 114-130 mmHg, while diastolic pressure ranged from 72-80 mmHg and 74-84 mmHg, respectively. Although differences were not statistically significant, Group 1 exhibited smoother trends, possibly reflecting better pain control (Figure 4).

**Figure 5:**
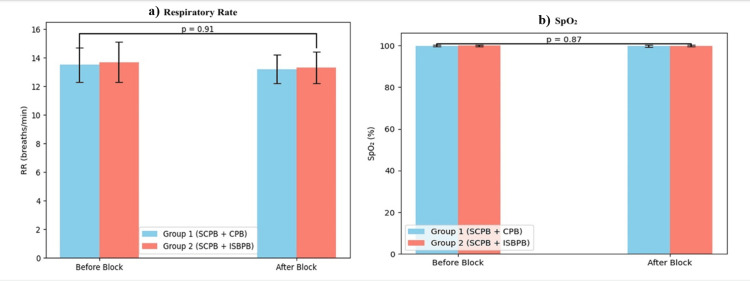
Comparison of respiratory rate and SpO2 between groups (N = 30). (a) Comparison of respiratory rate (breaths/min in n). (b) Comparison of SpO_2_ (in %). The independent samples t-test was used for respiratory parameters. P-value <0.05 is statistically significant.

Respiratory parameters also remained stable. Mean respiratory rate was 14-18 breaths/minute in both groups, and SpO₂ levels stayed above 96% throughout. No cases of hemidiaphragmatic paralysis or respiratory compromise occurred in Group 1 (Figure 5).

Adverse events were rare (n = 5) in both groups. Group 1 reported only minor local discomfort in two patients. Group 2 had three cases of transient hemidiaphragmatic paralysis for a few minutes and one instance of mild hoarseness, suggestive of recurrent laryngeal nerve involvement, all resolving without intervention. No cases of local anesthetic systemic toxicity, vascular puncture, or persistent neurological deficits occurred in either group.

## Discussion

The present prospective study compared the efficacy of two groups of ultrasound-guided regional anaesthetic techniques, i.e., SCPB+CPB and SCPB+ISBPB, for clavicle fixation. Our findings demonstrate that SCPB+CPB provides significantly longer analgesia, lower pain scores, reduced rescue analgesic requirements, and fewer complications than SCPB+ISBPB, supporting the growing evidence favoring fascial plane blocks as effective and safer alternatives to conventional brachial plexus approaches.

A key result of this study was the markedly prolonged analgesia observed with SCPB+CPB (mean 15 hours) compared with SCPB+ISBPB (10 hours), leading to delayed rescue analgesic demand and reduced opioid consumption. This aligns with observations by Zhuo et al. (2022), who reported comparable intraoperative anesthesia but superior safety when CPB was combined with cervical plexus block [11]. Similarly, Xu et al. (2023) demonstrated that SCPB combined with CPB provided effective surgical anesthesia with fewer complications, corroborating our findings [8].

The safety advantage of CPB is particularly relevant. Riazi et al. (2008) reported phrenic nerve palsy rates approaching 100% with high-volume ISBPB, a complication absent in our CPB group [12]. Continuous catheter techniques proposed to extend ISBPB duration remain technically complex and infection-prone, as noted by Singhal and Taksande (2024) [13]. In contrast, CPB achieved extended analgesia without respiratory compromise, highlighting its suitability for patients with pulmonary comorbidities.

The superior analgesic efficacy of CPB is anatomically plausible. Yoshimura and Morimoto (2020) demonstrated that key sensory nerves to the clavicle, namely, subclavian, suprascapular, long thoracic, and lateral pectoral, traverse the clavipectoral fascia, allowing effective blockade when local anesthetic is deposited in this plane. ISBPB, while producing dense blockade proximally, may not reliably cover all distal branches and risks unintended spread to the phrenic or recurrent laryngeal nerves [14].

Effective perioperative pain control is critical in clavicle surgery, as inadequate analgesia can impair respiratory function, delay mobilization, and predispose to chronic pain syndromes. Makkad and Kachulis (2024) highlighted these consequences, emphasising the importance of optimized analgesia within enhanced recovery after surgery protocols [15]. By prolonging analgesia and minimizing opioid use, SCPB+CPB aligns with global opioid-sparing initiatives, improves patient satisfaction, and may reduce healthcare burdens.

Respiratory safety is a major consideration in regional anaesthesia. Coviello et al. (2025) reported that ISBPB-associated hemidiaphragmatic paralysis can precipitate respiratory distress in patients with chronic lung disease [16]. Consistent with this, three patients in our ISBPB group exhibited transient diaphragmatic dysfunction, while none in the CPB group did. Moreover, complications such as recurrent laryngeal nerve palsy, Horner’s syndrome, and systemic toxicity were absent with CPB. The use of ultrasound likely contributed to these favorable outcomes by improving accuracy and reducing intravascular injection, as supported by Guay et al. (2019) [17].

The strengths of this study include its prospective randomized design, validated pain and hemodynamic measures, and clinically meaningful endpoints. Conducting the trial in a tertiary center enhanced its generalizability, as noted by Bodian et al. (2001) [18]. However, limitations include a modest sample size, exclusion of high-risk patients, short follow-up, and potential operator dependency, which may limit external applicability. As group allocation was based on anesthesiologist preference and clinical considerations rather than randomization, the possibility of selection bias cannot be excluded.

Future research should focus on larger multicentric trials, optimization of local anesthetic dosing as suggested by Surav et al. (2011) [19], evaluation of adjuvant use, long-term pain outcomes, and cost-effectiveness analyses, as recommended by Michelly Gonçalves Brandão et al. (2023) [20].

## Conclusions

This study provides compelling evidence that SCPB+CPB is a superior alternative to SCPB+ISBPB for clavicle fixation, offering extended analgesia, fewer complications, and a strong opioid-sparing effect. As ultrasound-guided fascial plane blocks continue to evolve, CPB is poised to become a key component of multimodal analgesic strategies in clavicle surgery. Within the limitations of a prospective observational design, the findings suggest that SCPB+CPB is associated with longer postoperative analgesia and fewer respiratory complications compared with SCPB+ISBPB in clavicle fixation surgery.

## Appendices

**Table 1:** Study questionnaire.

Name:									
Age:									
MRD no.:									
BMI:									
Address:									
Socioeconomic status:									
Diagnosis:									
Proposed surgery:									
ASA grading:									
H/O local anaesthetic allergy:									
Intraoperative vitals every 15 minutes									
Time		SpO2		Blood pressure		Heart rate		Respiratory rate	
Postoperative monitoring									
Time	Immediately after the procedure (0 minutes)		30 minutes	4 hours	8 hours	12 hours	18 hours		24 hours
Pain (VAS)									
Rescue analgesic consumption									
Time of first rescue analgesic									
Total opioid consumption in the first 12 to 24 hours									
